# “Ablate and Pace” with Conduction System Pacing: Concomitant versus Delayed Atrioventricular Junction Ablation

**DOI:** 10.3390/jcm13082157

**Published:** 2024-04-09

**Authors:** Pietro Palmisano, Matteo Ziacchi, Gabriele Dell’Era, Paolo Donateo, Lorenzo Bartoli, Giuseppe Patti, Jacopo Senes, Antonio Parlavecchio, Mauro Biffi, Michele Accogli, Giovanni Coluccia

**Affiliations:** 1 Cardiology Unit, “Card. G. Panico” Hospital, 73039 Tricase, Italy; 2 Institute of Cardiology, University of Bologna, S. Orsola-Malpighi University Hospital, 40126 Bologna, Italy; 3 Division of Cardiology, University of Eastern Piedmont, Maggiore della Carità Hospital, 28100 Novara, Italy; 4 Department of Cardiology, Arrhythmology Center, ASL 4 Chiavarese, 16033 Lavagna, Italy; paolo.donateo@gmail.com (P.D.);; 5 Cardiology Unit, Department of Clinical and Experimental Medicine, University of Messina, 98122 Messina, Italy

**Keywords:** atrial fibrillation, conduction system pacing, His bundle pacing, left bundle branch area pacing, catheter ablation, AV junction ablation, ablate and pace

## Abstract

**Objectives:** Conduction system pacing (CSP) and atrioventricular junction ablation (AVJA) improve the outcomes in patients with symptomatic, refractory atrial fibrillation (AF). In this setting, AVJA can be performed simultaneously with implantation or in a second procedure a few weeks after implantation. Comparison data on these two alternative strategies are lacking. **Methods:** A prospective, multicentre, observational study enrolled consecutive patients with symptomatic, refractory AF undergoing CSP and AVJA performed in a single procedure or in two separate procedures. Data on the long-term outcomes and healthcare resource utilization were prospectively collected. **Results:** A total of 147 patients were enrolled: for 105 patients, CSP implantation and AVJA were performed simultaneously (concomitant AVJA); in 42, AVJA was performed in a second procedure, with a mean of 28.8 ± 19.3 days from implantation (delayed AVJA). After a mean follow-up of 12 months, the rate of procedure-related complications was similar in both groups (3.8% vs. 2.4%; *p* = 0.666). Concomitant AVJA was associated with a lower number of procedure-related hospitalizations per patient (1.0 ± 0.1 vs. 2.0 ± 0.3; *p* < 0.001) and with a lower number of hospital treatment days per patient (4.7 ± 1.8 vs. 7.4 ± 1.9; *p* < 0.001). **Conclusions:** Concomitant AVJA resulted as being as safe as delayed AVJA and was associated with a lower utilization of healthcare resources.

## 1. Introduction

In patients with symptomatic refractory atrial fibrillation (AF) and uncontrolled, drug-refractory ventricular rate, permanent pacemaker (PM) implantation followed by atrioventricular junction ablation (AVJA) (“ablate and pace”, A&P, strategy) is effective in controlling the symptoms and improving quality of life. To avoid the risk of developing pace-induced cardiomyopathy, which can occur in 10–20% of patients with a high burden of right ventricular (RV)-only pacing [1,2,3], biventricular pacing (BVP) has been proposed as an alternative to RV pacing [4]. In these patients, BVP significantly improves their symptoms, functional capacity, and left ventricular (LV) function in comparison with RV pacing alone [4] and proved superior to conventional medical therapy in reducing hospitalization for heart failure (HF) and all-cause mortality [5,6,7].

However, BVP delivers non-physiological ventricular activation because it does not recruit the conduction system. When delivered to patients with a narrow QRS, it prolongs ventricular activation [8]. Conduction system pacing (CSP) using His bundle pacing (HBP) or left bundle branch area pacing (LBBAP) has been proposed as an alternative to BVP in patients undergoing AVJA [9,10,11,12]. This pacing modality can be delivered with a single lead, recruits the intrinsic conduction system, and, therefore, may preserve physiological ventricular activation after AVJA.

Given the potential risk of lead malfunction (mainly related to dislodgement) during the first weeks after device implantation in patients becoming PM-dependent after AVJA, ablation is routinely performed 4–6 weeks after device implantation to allow lead stabilization [6,13]. However, previous studies enrolling patients undergoing A&P with CSP have shown that performing AVJA simultaneously with the implantation procedure is feasible and safe [9,10,11,12,14,15,16,17,18,19,20]. This approach is potentially advantageous as it avoids the need for a new hospitalization, and allows the benefits of the A&P strategy to be provided already at the time of implantation. However, data comparing A&P performed with a single-procedure approach versus two separate procedures are lacking.

We conducted a prospective observational study enrolling consecutive patient candidates for A&P, aiming to compare the feasibility and the long-term safety of a strategy of AVJA performed simultaneously with CSP implantation with a strategy of AVJA performed later, in a second procedure.

## 2. Materials and Methods

### 2.1. Study Design and Participants

The POINTED (Impact on Patient Outcome and Healthcare Utilization of Cardiac ImplaNTble Electronic Devices Complications) registry is a prospective, multicentre, observational study designed to collect data on the long-term outcome of patients undergoing cardiac implantable electronic device implantation [10]. The study was conducted in accordance with the Declaration of Helsinki. The protocol was approved by the ethics committee of the participating institutions, and the study has been registered in a public database (clinicaltrials.gov under identifier NCT03612635). All patients provided their written informed consent to participate in the registry.

The study population consisted of consecutive patients undergoing the A&P strategy with CSP (regardless of the timing in which AVJA was performed) for symptomatic, refractory AF with an uncontrolled ventricular rate in the period from January 2021 to May 2023 at four Italian high-volume arrhythmia centers. Patients with a device with CSP and who were not primarily candidates for A&P, in whom the need for AVJA arose following implantation, were excluded from the analysis. A small number of patients in which the procedure was performed, with the guidance of an electroanatomical mapping system, were also excluded [21].

The primary aim of the study was to compare the procedural aspects and long-term outcomes (including the risk of procedure-related complications, procedure-related hospitalizations, hospitalizations due to HF, AVJA outcome, and pacing parameters) of a strategy of CSP and AVJA performed simultaneously in the same procedure (concomitant AVJA), with a strategy of CSP and AVJA performed in two separate procedures (delayed AVJA). The secondary aim was to compare the impact on healthcare resource utilization of these two alternative strategies.

### 2.2. Procedures

Indication for the A&P strategy was based on the current guidelines for AF management [13]. According to the current guidelines on the prevention of sudden cardiac death [22], the device implanted was a PM or an implantable defibrillator. All procedures were performed by electrophysiologists with extensive experience both in CSP and in catheter ablation. At the time of A&P, data on the baseline characteristics, clinical indication, type of device implanted, and procedural details, including procedure times, pacing parameters, AVJA acute success rate, and intra-procedural complications, were collected.

The choice between HBP and LBBAP was based on the operator’s preference and the patient’s characteristics. In general, HBP was preferred over LBBAP in patients with a baseline narrow QRS. An RV backup lead was implanted in all HBP patients [23]—and optionally in LBBAP patients—based on the operator’s preference. The atrial lead was optionally implanted based on the operator’s preference and the patient’s characteristics. The methods previously described for HBP lead implantation [24], LBBAP lead implantation, and conduction system capture confirmation [24,25] were used.

AVJA was performed at the time of the device implantation or at a later date, depending on the patient’s clinical circumstances and physician’s discretion. When the AVJA was scheduled in a second procedure (delayed AVJA), it was usually performed after the second outpatient check of the device (which was performed, on average, 2 weeks after implantation) if the stability of the electrical parameters of the CSP lead was confirmed.

AVJA was performed with radiofrequency energy, by means of a conventional right-sided approach via the femoral vein. A left-sided approach via the femoral artery was used if right-sided ablation failed to achieve persistent third-degree AV block [7]. 

In the right-sided approach, the ablation catheter (a standard D-curve, 4 mm, non-irrigated tip catheter from different manufacturers) was placed directly at the AV nodal region (small far-field His bundle and large atrial electrograms), and ablation was performed in temperature control (50 to 60 °C) at a maximum power of 50 W for a total duration of 60 s, aiming to achieve complete AV block. Additional lesions were performed at the operator’s discretion. In the case of a left-sided approach, a standard D-curve, 4/3.5 mm, irrigated tip catheter (from different manufacturers) was placed directly at the proximal His bundle site, and ablation was performed in temperature control (43 °C) at a maximum power of 35 W for a total duration of 60 s, aiming to achieve complete AV block. In the patients in which HBP and AVJA were concomitant, the device pocket was closed after having performed ablation, to allow for HBP lead repositioning in the case of a relevant increase in its capture threshold following ablation. 

After AVJA, the final programming of the implanted device was based on the available evidence [26,27]. Specifically, in the acute phase, the device was programmed with a lower rate limit of 80–100 b.p.m., according to the baseline heart rate. Within the first month after the AVJA, the lower rate limit was gradually reduced to 70 b.p.m. in all patients. During follow-up, when a recovery of AV conduction was detected, a repeat ablation was performed.

### 2.3. Follow-Up

Follow-up observation started at the time of the device implantation. All patients were evaluated at 1, 2, 4, and 12 weeks after A&P and then regularly at 6-month intervals or more frequently when clinically indicated. Each scheduled or unscheduled outpatient clinic visit included the following: adverse event assessment, including procedure-related complications, procedure-related hospitalizations, and hospitalizations due to HF (all defined below); medical therapy assessment; physical examination (including pocket and femoral venous access site assessment); surface standard electrocardiogram; echocardiogram; device interrogation, including an assessment of the electrical parameters of the CSP lead, and of the persistence of AV block. If patients missed a scheduled in-hospital follow-up visit, they or their relatives were contacted by phone to determine their survival status, and to assess any adverse events. 

Procedure-related hospitalization was defined as any hospitalizations related to the device implantation, AVJA procedure, redo AVJA, and procedure-related complications management.

Hospitalization due to HF was defined as an overnight stay, or longer, in a hospital environment (emergency room, observation unit, in-patient care, or similar facility, including admission to a daycare facility) due to HF as the primary reason for hospitalization.

During the follow-up, data on the occurrence of procedure-related complications (defined below), refs. [10,16,21,25,28,29,30], procedure-related hospitalizations, AVJA outcomes, and pacing parameters were prospectively recorded in a secure electronic data management system.

### 2.4. Definition of Procedure-Related Complications

Procedure-related complications were defined as any adverse event related to implantation and AVJA, intra- or post-procedural, including cardiac tamponade, device infection (systemic or local infection), device malfunction, pneumothorax, pocket hematoma, lead dislodgement, lead failure, and CSP lead deactivation. CSP lead deactivation was defined as the deactivation of the lead due to loss of capture, excessive increase of capture threshold (leading to a premature battery depletion), or phrenic nerve stimulation irresolvable by reprogramming [10,16].

### 2.5. Statistical Analysis

Descriptive statistics were reported as means ± standard deviations for normally distributed continuous variables and were compared by means of the Student’s *t*-test and analysis of variance. Continuous variables with skewed distribution were reported as medians with an interquartile range (IQR) and compared by means of the Mann–Whitney U test. Categorical data were expressed as percentages, reported in contingency tables, and compared by means of the χ^2^ test or Fisher’s exact test, as appropriate. Event and event-free curves were based on Kaplan–Meier analyses, stratified by study group, and compared by means of the log-rank test. The cumulative probability of an event was estimated with its standard error. The effect of individual variables on the risk of procedure-related complications was investigated by using univariate Cox proportional hazards models applied to the whole study population. The variables that showed an effect on the risk of complications with a significance level of <0.2 on the univariate analyses were entered into multivariable Cox proportional hazard models. Cox model findings are presented as hazard ratios, tests of significance, and 95% confidence intervals. Interactions between the covariates were tested for significance in the model. *P*-values of less than 0.05 were considered statistically significant. The data were analyzed by means of the statistical software package Statistica version 6.1 (StatSoft Inc., Tulsa, OK, USA).

## 3. Results

### 3.1. Baseline Characteristics and Procedural Findings

During the study period, a total of 147 consecutive patients were enrolled: in 105 (71.4%), AVJA was performed at the time of device implantation (concomitant AVJA group), and in 42 (28.6%), in a second procedure (delayed AVJA group), with a mean of 28.8 ± 19.3 days (range: 3–86) from implantation. In three patients of the delayed AVJA group (7.1%), AVJA was performed earlier than scheduled (a mean of 9.0 ± 5.6 days after implantation) due to their deteriorating clinical status, leading to hospitalization for HF.

Table 1 compares the baseline characteristics of the two study groups. Compared to the delayed AVJA patients, concomitant AVJA patients had a significantly worse NYHA class and, more frequently, had permanent rather than persistent or paroxysmal AF. The other characteristics were similar between the two groups.

In Table 2 the procedural findings are detailed and compared between the two groups. At the time of implantation, LBBAP was successfully performed in a total of 74 patients (50.3%), whereas 73 patients (49.7%) underwent successful HBP. Among patients with LBBAP, prior attempts at HBP had failed in three (4.1%) patients, while in two (2.7%) of the HBP patients, prior attempts at LBBAP had failed.

Compared to the delayed AVJA patients, concomitant AVJA patients received HBP more frequently than LBBAP; they also received a biventricular PM more frequently; in addition, in these patients, the atrial lead was implanted less frequently, and the RV backup lead more frequently, particularly in the subgroup of patients receiving LBBAP. Consequently, concomitant AVJA patients received a mean total number of ventricular leads per patient significantly higher than delayed AVJA patients.

No significant differences in the acute pacing parameters, as well as in the AVJA procedure findings were observed between the two groups (all *p*-values for comparisons > 0.05).

### 3.2. Follow-Up

The median duration of follow-up was 12 months (IQR: 5–17) and was similar between the two study groups (*p* = 0.381). The pacing parameters, outcome of AVJA, and data on procedure-related complications and hospitalizations at follow-up are detailed in Table 3. 

On the last follow-up, both HBP and LBBAP leads maintained a capture threshold, both in voltage and in pulse width stability, compared to the implantation, with no differences between the two groups. Three months after implantation (at the end of the stabilization phase), the capture thresholds remained low and stable compared to the implantation in all LBBAP patients of both groups in whom a biventricular device with a backup RV lead was implanted (*n* = 34), and backup pacing was turned off in order to maximize the battery’s longevity.

**Figure 1:**
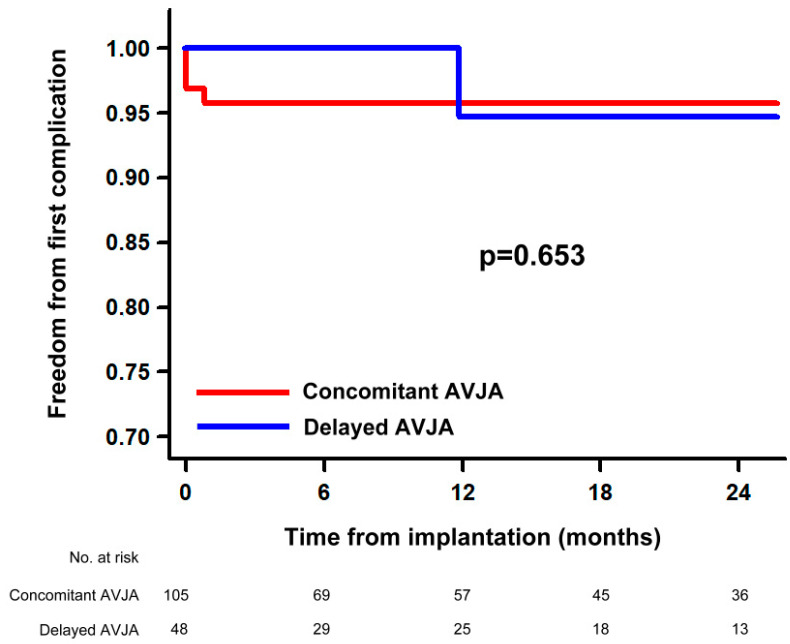
Twenty-four-month cumulative free survival from first complication: comparison between the two study groups. AVJA: atrioventricular junction ablation.

The rate of procedure-related complications was similar in both groups (3.8% in the concomitant AVJA group vs. 2.4% in the delayed AVJA group; *p* = 0.666; Table 3). The 24-month cumulative free survival from first procedure-related complications was similar in the two groups (Figure 1). In both univariate and multivariate analyses, no clinical and procedural variables (including AVJA strategy) were significantly associated with a higher risk of procedure-related complications.

Details on the complications are reported in Table 3. In the concomitant AVJA group, we observed a case of intra-procedural acute increase in the HBP threshold and a case of intra-procedural LBBAP lead dislodgement, both occurring during AVJA. In both cases, the CSP leads were successfully repositioned without clinical consequences. In this group, we also observed a single case of groin hematoma, treated conservatively, resulting in an extension of hospitalization by 2 days, and a case of backup RV lead dislodgment into the superior vena cava (causing diaphragmatic stimulation) in a patient with twiddler syndrome, occurring 3 months after implantation. In the delayed AVJA group, we observed only a case of late increase in the HBP threshold, requiring lead repositioning, detected 11 months after implantation. No adverse events related to a loss of CSP lead capture were observed in both groups during follow-up. No device-related infections were observed in both groups.

The rate of AV node conduction recovery during follow-up was similar in both groups (5.7 vs. 4.8%; *p* = 0.818). 

During follow-up, six patients (4.1%) were hospitalized for HF: two (1.9%) in the concomitant AVJA group and four (9.5%) in the delayed AVJA group (*p* = 0.035). Three of the four patients of the delayed AVJA group were hospitalized due to their worsening clinical status while awaiting AVJA. In all three patients, AVJA was performed during hospitalization as a rescue treatment.

During follow-up, a significant increase in left ventricular ejection fraction (LVEF) was observed in the overall study population: from 43.3 ± 11.3% at baseline to 48.4 ± 10.6% during the follow-up (*p* < 0.001). The extent of LVEF improvement was similar in both study groups: 4.9 ± 6.7% vs. 4.6 ± 8.3% (*p* = 0.820).

### 3.3. Impact of Timing of Atrioventricular Junction Ablation on Healthcare Resources Utilization

**Figure 2:**
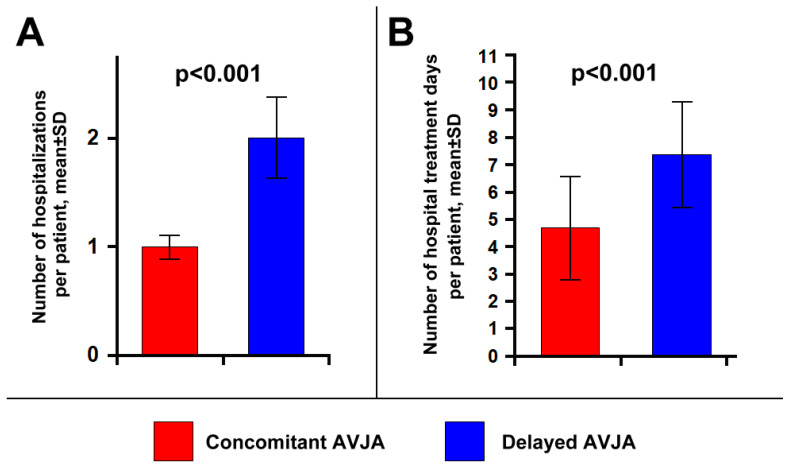
Comparison of the impact on healthcare utilization of “ablate and pace” with concomitant versus delayed atrioventricular junction ablation (AVJA): mean number of hospitalizations per patient (**A**); mean number of hospital treatment days per patient (**B**). The count included hospitalizations and hospital treatment days related to device implantation, AVJA procedure, redo AVJA, and complications management. AVJA: atrioventricular junction ablation; SD: standard deviation.

Compared to the strategy of delayed AVJA, the single-procedure approach was associated with a significantly lower number of hospitalizations per patient related to A&P (including hospitalizations related to device implantation, AVJA procedure, redo AVJA, and complications management) (1.0 ± 0.1 vs. 2.0 ± 0.3; *p* < 0.001; Figure 2A), and with a significantly lower number of hospital treatment days per patient (4.7 ± 1.8 vs. 7.4 ± 1.9; *p* < 0.001; Figure 2B). Excluding from the analysis the hospitalizations related to the AVJA procedure in the delayed AVJA group, the mean number of hospitalizations per patient related to A&P remained significantly higher in the delayed AVJA group compared to the concomitant AVJA group (1.1 ± 0.3 vs. 1.0 ± 0.1; *p* = 0.003).

## 4. Discussion

Our prospective, multicentre, observational study compared a strategy of CSP and AVJA performed simultaneously in the same procedure with a strategy of CSP and AVJA performed in two separate procedures in patients with symptomatic, drug-refractory AF and uncontrolled, high ventricular rates who underwent A&P. The main findings of this study are that the single-procedure strategy appears to have a safety profile comparable to the two-procedure strategy (specifically, in our experience, no patient in both study groups experienced adverse events related to a loss of CSP lead capture in the stabilization phase), and offers the advantage of significantly reducing the number of hospitalizations related to the A&P procedure, and consequently the number of hospital treatment days.

A&P routinely consists of two separate procedures: the device implantation and, subsequently, the AVJA. Many operators prefer to perform ablation 3–6 weeks after implantation to allow lead stabilization [5,6,7]. Given the low reported lead dislodgement rates in CSP, and especially in LBBAP [17,18,25,29], and given the possibility of implanting a backup lead for the sake of safety (especially in HBP), several operators routinely perform CSP implantation and AVJA simultaneously in the same procedure. This strategy has been evaluated in several studies, showing that it is feasible and safe [14,15,16,17,18,19,20]. However, no previous study has compared the single-procedure strategy with the two-procedure one. In our study, the risk of procedure-related complications of the two strategies was similar. Although we observed in the concomitant AVJA group two cases of intra-procedural CSP lead malfunction occurring during AVJA, these events had no significant clinical consequences. Of note, we have not observed any cases of lead dislodgement in the stabilization phase. These findings suggest that the single-procedure strategy could be as safe as the two-procedure strategy.

In our study, the strategy of concomitant AVJA was associated with a halving of A&P procedure-related hospitalizations with a consequent reduction in the number of hospitalization days per patient. This is not surprising, as this approach avoids the need for a new hospitalization for the ablation procedure. These findings could have interesting implications, as they suggest that this strategy can lead to a reduction in hospitalization-related risks for the patients, as well as an optimization in the use of healthcare resources and significant economic savings.

The strategy of delayed AVJA was associated with a significantly higher risk of hospitalizations for HF during follow-up. In the majority of patients of the delayed AVJA group, HF hospitalizations occurred while they were awaiting AVJA due to worsening clinical status. In these patients, ablation was performed during HF hospitalization as a rescue treatment. It is possible that the single-procedure strategy, allowing to provide the benefits of A&P already at the time of implantation, prevents further clinical deterioration of patients, potentially reducing the risk of hospitalizations for HF.

Due to the non-randomized, observational design of the study, the two study groups showed significant differences in some baseline characteristics. Specifically, patients in the delayed AVJA group had fewer symptoms (specifically, they had a significantly better NYHA class) and, more frequently, had intermittent forms of AF. This likely reflects the fact that physicians used the two-procedure strategy more often in the more clinically stable patients, in whom ablation could be delayed.

Some differences were also observed in the procedural findings. In the patients of the delayed AVJA group, HBP was used less than LBBAP. AVJA can be particularly tricky in the presence of an HBP lead, as ablation near the distal electrode can result in a significant rise in the His bundle capture threshold, which may require lead repositioning. If this occurs at the time of the implantation procedure, as the device pocket is kept open until ablation is completed, the lead repositioning has potentially no clinical impact, as it does not require a delayed surgical revision of the pacing system. There is also a theoretical risk of a rise in His bundle capture threshold occurring in the first few days after AVJA as a consequence of a late growth of radiofrequency ablation lesions involving the pacing site of the HBP lead. In LBBAP, on the other hand, the risk of an acute increase in the capture threshold during ablation is lower because the ablation site is more distant from the pacing site. Probably, this was one of the reasons why HBP was an underused pacing modality in patients in which a delayed AVJA was planned.

In delayed AVJA patients, the RV backup lead was implanted more rarely than in concomitant AVJA patients. This impacted the total number of ventricular leads implanted for each patient, as well as the complexity of the implanted devices (lower rate of biventricular devices), and was likely due to the fact that performing AVJA a few weeks post-implantation, after the lead stabilization phase, substantially reduced the potential risks related to lead malfunction in patients becoming PM-dependent, making the RV backup lead unnecessary. These findings have significant clinical implications as, as shown in several studies [29,31,32,33,34,35], the implantation of fewer leads and of less complex devices reduces the risk of infection and lead-related complications, as well as reducing the cost of the procedure. On the other hand, we observed that, during follow-up, in all study populations, CSP thresholds remained stable, and no patient in either study group experienced adverse events related to the loss of capture occurring during the stabilization phase. Also of note, after the stabilization phase, the RV backup lead was deactivated in all LBBAP patients in both groups, as the capture thresholds remained stable. These findings could suggest that, especially in patients receiving LBBAP, which, as known, shows lower and stable capture thresholds during the follow-up compared to HBP [17,18,25,29,31,32,33,34,35], the RV backup lead could be superfluous, even in patients in whom AVJA is performed simultaneously with the implantation.

In our study, the number of LBBAP patients receiving an RV backup lead was significantly higher than in the previous studies. This is probably due to the fact that many operators involved in our study preferred to implant the RV backup lead in patients treated with LBBAP in whom AVJA was performed at the time of implantation in order to minimize the risk of potential consequences related to LBBAP lead dislodgement occurring in the acute phase in patients without a stable escape rhythm after AVJA. This strategy was not supported by the safety data available in the literature [10,17,18,19] (confirmed by our follow-up findings), suggesting that an RV backup lead in patients undergoing LBBAP is mostly unnecessary. The risk of potential adverse events related to the implantation of an additional RV lead (including worsening of tricuspid regurgitation and increased risk of infection) is certainly higher than the potential risks related to LBBAP lead dislodgement in PM-dependent patients. 

As recommended in the current guidelines [23], all HBP patients undergoing AVJA received an RV backup lead. The presence of a second ventricular lead, in addition to increasing the risk of complications, may increase the risk of pace-induced cardiomyopathy as a result of RV-only pacing if a chronic increase in the HBP threshold occurs.

Compared with LBBAP, the mean pacing threshold of HBP tended to increase during the follow-up (from 1.3 mV at implant to 1.5 mV at the last follow-up). This finding was in agreement with the results of previous studies showing a non-negligible long-term complication rate of HBP being related to increases in pacing thresholds and the need for lead revision [29,36,37]. 

In our study, a high proportion of patients received an atrial lead, even though the vast majority of patients had permanent AF. This choice by the operators was probably due to the availability of clinical evidence showing that spontaneous sinus rhythm restoration can be observed during the follow-up in a non-negligible rate of patients with AF considered as “permanent”, treated with the A&P strategy. However, further evidence is needed to clarify whether the potential benefit of the atrial lead implantation in these patients balances the increased risk of complications resulting from the implantation of an additional lead and of a more complex pacing system.

### Study Limitations

The main limitations of this study were its observational, non-randomized design and the small size of the sample. Due to the non-randomized design of the study, we found significant differences in some baseline characteristics of the two study groups. As confounding factors and selection bias may not be excluded, our results are not conclusive and should be interpreted with caution. 

As our study was observational, the decision to perform AVJA during device implantation or in a subsequent procedure was left to the operator’s discretion and was not driven by the study. The choice between the two strategies was probably partly influenced by the patient’s characteristics. Consequently, the two study groups, in addition to having significant differences in some baseline characteristics and a dissimilar number of participants, received a different type of CSP. Specifically, the concomitant AVJA group had a higher number of patients receiving HBP, while the delayed AVJA group had a higher number of patients receiving LBBAP. These differences may have impacted the outcome results of the study.

In our study, we observed no significant difference in the risk of procedure-related complications in the two study groups. We cannot exclude that the failure to find a statistically significant difference between the two groups was due to an undersizing of the sample, the short follow-up, and the low number of events. For these reasons, further prospective, randomized, larger population studies are needed to confirm our findings.

In this study, HBP and LBBAP were analyzed together. These two pacing modalities are very different regarding the risk of complications and the complexity of AVJA [10,29,30]. These differences may have affected the outcomes.

Due to the observational nature of this study, in patients in whom AVJA was performed in a second procedure, the precise timing for ablation was not predetermined and depended on several factors, including hospital organizational aspects, the availability of the patient for a second hospitalization, and the clinical status of the patients. Consequently, the timing of the ablation was very broad.

## 5. Conclusions

The results of this multicentre, prospective, observational study suggest that in candidates for A&P, performing CSP and AVJA simultaneously during the same procedure could be as feasible and safe as performing CSP and AVJA in two separate procedures. The single-procedure strategy could significantly reduce the number of hospitalizations and days of hospitalization per patient related to A&P and could be associated with a lower risk of HF hospitalizations.

Due to the methodological limitations of the present study, additional randomized clinical studies are needed to confirm these findings.

## Figures and Tables

**Table 1:** Baseline characteristics: comparison between concomitant AVJA group and delayed AVJA group.

Baseline Characteristics	Concomitant AVJA (*n* = 105)	Delayed AVJA (*n* = 42)	*p*-Value
Male, *n* (%)	48 (45.7)	14 (33.3)	0.170
Age in years, mean ± SD	77.1 ± 9.2	76.6 ± 8.6	0.762
NYHA class, mean ± SD	2.9 ± 0.7	2.6 ± 0.6	**0.016**
Class I, *n* (%)	2 (1.9)	1 (2.4)	0.854
Class II, *n* (%)	23 (21.9)	16 (38.1)	**0.045**
Class III, *n* (%)	59 (56.2)	23 (54.8)	0.875
Class IV, *n* (%)	21 (20.0)	2 (4.8)	**0.022**
LVEF in %, mean ± SD	42.3 ± 11.8	45.4 ± 10.1	0.137
LVEF < 40%, *n* (%)	46 (43.8)	23 (54.8)	0.229
History of AF			
Permanent, *n* (%)	97 (92.4)	30 (71.4)	**0.001**
Persistent, *n* (%)	5 (4.8)	7 (16.7)	**0.017**
Paroxysmal, *n* (%)	3 (2.9)	5 (11.9)	**0.029**
Duration of AF in months, median (IQR)	12.6 ± 7.8	12.1 ± 8.6	0.734
Previous electrical cardioversion/s, *n* (%)	63 (60.0)	28 (66.7)	0.452
Previous attempt/s at catheter ablation of AF, *n* (%)	13 (12.4)	7 (16.7)	0.494
No. of hospitalizations for HF in the previous year, mean ± SD	1.7 ± 1.1	1.5 ± 1.8	0.414
Standard electrocardiogram at implantation			
Heart rate in b.p.m., mean ± SD	107.2 ± 25.0	109.6 ± 26.7	0.659
QRS width in ms, mean ± SD	105.0 ± 30.8	110.6 ± 26.6	0.303
QRS width > 130 ms, *n* (%)	37 (35.2)	17 (40.5)	0.552
LBBB morphology, *n* (%)	22 (21.0)	14 (33.3)	0.115
RBBB morphology, *n* (%)	9 (8.6)	1 (2.4)	0.178
IVCD morphology, *n* (%)	6 (5.7)	2 (4.8)	0.818
CHA_2_DS_2_-VASc score, mean ± SD	5.1 ± 1.8	4.7 ± 2.1	0.248
**Associated disorders**			
Hypertension on therapy, *n* (%)	88 (83.8)	36 (85.7)	0.774
Dyslipidemia, *n* (%)	57 (54.3)	20 (47.6)	0.465
Diabetes, *n* (%)	33 (31.4)	8 (19.0)	0.131
Obesity, *n* (%)	42 (40.0)	13 (31.0)	0.306
CAD, *n* (%)	14 (13.3)	5 (11.9)	0.816
Previous PCI (*n*, %)	12 (11.4)	4 (9.5)	0.738
Valvular heart disease, *n* (%)	12 (11.4)	7 (16.7)	0.392
Valve prosthesis, *n* (%)	6 (5.7)	1 (2.4)	0.391
Previous TIA/stroke, *n* (%)	15 (14.3)	7 (16.7)	0.715
Chronic renal failure, *n* (%)	47 (44.8)	16 (38.1)	0.461
COPD, *n* (%)	27 (25.7)	6 (14.3)	0.134

AF: atrial fibrillation; AVJA: atrioventricular junction ablation; CAD: coronary artery disease; COPD: chronic obstructive pulmonary disease; HF: heart failure; IQR: interquartile range; IVCD: non-specific intraventricular conduction disturbance; LBBB: left bundle branch block; LVEF: left ventricular ejection fraction; NYHA: New York Heart Association; PCI: percutaneous coronary intervention; RBBB: right bundle branch block; SD: standard deviation; TIA: transient ischemic attack.

**Table 2:** Procedural findings: comparison between concomitant AVJA group and delayed AVJA group.

Parameters	Concomitant AVJA (*n* = 105)	Delayed AVJA (*n* = 42)	*p*-Value
**Implantation**			
Procedure time in minutes, mean + SD	65.9 ± 25.6	63.1 ± 27.0	0.556
Fluoroscopy duration in minutes, mean ± SD	10.2 ± 7.8	8.5 ± 5.6	0.201
Pacing modality			
HBP, *n* (%)	64 (61.0)	9 (21.4)	**<0.001**
LBBAP, *n* (%)	41 (39.0)	33 (78.6)	**<0.001**
Type of device implanted			
Single chamber PM, *n* (%)	3 (2.9)	1 (2.4)	0.873
Dual-chamber PM, *n* (%)	35 (33.3)	21 (50.0)	0.060
Biventricular PM, *n* (%)	43 (41.0)	9 (21.4)	**0.025**
Dual-chamber ICD, *n* (%)	11 (10.5)	2 (4.8)	0.270
Biventricular ICD, *n* (%)	13 (12.4)	9 (21.4)	0.165
Atrial lead implanted, *n* (%)	49 (46.7)	36 (85.7)	**<0.001**
RV backup lead implanted, *n* (%)	98 (93.3)	23 (54.8)	**<0.001**
Among patients receiving HBP, *n*/*n* (%)	64/64 (100.0)	9/9 (100.0)	1.000
Among patients receiving LBBAP, *n*/*n* (%)	35/41 (85.4)	14/33 (42.4)	**<0.001**
Total number of ventricular leads implanted per patient, mean ± SD	1.9 ± 0.2	1.0 ± 0.5	**<0.001**
Pacing parameters of CSP lead at implantation			
HBP	*n* = 64	*n* = 9	
Capture threshold in Volt, mean ± SD	1.3 ± 0.7	1.4 ± 0.8	0.694
Pulse width in ms, mean ± SD	1.0 ± 0.1	1.0 ± 0.0	1.000
Pacing impedance in Ohm, mean ± SD	568.6 ± 134.2	535.6 ± 141.7	0.495
LBBAP	*n* = 41	*n* = 33	
Capture threshold in Volt, mean ± SD	0.5 ± 0.4	0.6 ± 0.5	0.342
Pulse width in ms, mean ± SD	0.5 ± 0.0	0.5 ± 0.0	1.000
Pacing impedance in Ohm, mean ± SD	666.7 ± 131.2	648.3 ± 183.2	0.617
**AVJA**			
Procedure time in minutes, mean + SD	27.9 ± 13.4	26.6 ± 15.7	0.614
Fluoroscopy duration in minutes, mean ± SD	2.6 ± 3.2	3.2 ± 3.1	0.302
Number of ablation lesions, mean ± SD	2.8 ± 2.3	2.5 ± 1.9	0.455
Ablation duration in minutes, mean ± SD	2.8 ± 2.8	2.2 ± 1.5	0.191
Acute success, *n* (%)	103 (98.1)	42 (100.0)	0.368
Presence of a stable escape rhythm after AVJA, *n* (%)	41 (39.0)	18 (42.9)	0.670
Paced QRS width after AVJA in ms, mean ± SD	108.2 ± 17.3	113.2 ± 22.3	0.148

AVJA: atrioventricular junction ablation; CSP: conduction system pacing; ICD: implantable cardioverter-defibrillator; HBP: His bundle pacing; LBBAP: left bundle branch area pacing; PM: pacemaker; RV: right ventricular; SD: standard deviation.

**Table 3:** Outcomes at last follow-up: comparison between concomitant AVJA group and delayed AVJA group.

Parameters	Concomitant AVJA (*n* = 105)	Delayed AVJA (*n* = 42)	*p*-Value
**Pacing parameters of CSP lead**			
HBP	*n* = 64	*n* = 9	
Capture threshold in Volt, mean ± SD	1.5 ± 1.2 *	1.6 ± 1.6 *	0.823
Pulse width in ms, mean ± SD	1.0 ± 0.1 *	1.0 ± 0.0 *	1.000
Pacing impedance in Ohm, mean ± SD	465.3 ± 232.3 *	484.5 ± 229.7 *	0.817
LBBAP	*n* = 45	*n* = 40	
Capture threshold in Volt, mean ± SD	0.5 ± 0.7 *	0.6 ± 0.5 *	0.456
Pulse width in ms, mean ± SD	0.5 ± 0.0 *	0.5 ± 0.0 *	1.000
Pacing impedance in Ohm, mean ± SD	644.3 ± 189.3 *	658.9 ± 202.6 *	0.732
Paced QRS width in ms, mean ± SD	110.26 ± 16.9	112.5 ± 19.7	0.738
**Outcome of AVJA**			
Repeated ablation due to regression of AV block, *n* (%)	6 (5.7)	2 (4.8)	0.818
Time interval between the first and second ablation in days, mean ± SD	36.3 ± 22.5	28.2 ± 27.1	0.065
**Complications**			
Patients with at least one complication, *n* (%)	4 (3.8)	1 (2.4)	0.666
Intra-procedural complications			
Acute increase in HBP threshold occurring during AVJA, *n* (%)	1 (1.0)	0 (0)	0.526
LBBAP lead dislodgement occurring during AVJA, *n* (%)	1 (1.0)	0 (0)	0.526
Vascular access site complications, *n* (%)	1 (1.0)	0 (0)	0.526
Late complications			
High voltage lead dislodgement, *n* (%)	1 (1.0)	0 (0)	0.526
HBP lead repositioning for increase in threshold, *n* (%)	0 (0)	1 (2.4)	0.113
**Impact of “ablate and pace” on healthcare utilization ^§^**			
Number of procedure-related hospitalizations per patient, mean ± SD	1.0 ± 0.1	2.0 ± 0.3	**<0.001**
Number of hospital treatment days per patient, mean ± SD	4.7 ± 1.8	7.4 ± 1.9	**<0.001**

* *p* > 0.05 compared with implantation. ^§^ The count included hospitalizations and hospital treatment days related to device implantation, AVJA procedure, redo AVJA, and complications management. AV: atrioventricular; AVJA: atrioventricular junction ablation; CSP: conduction system pacing; HBP: His bundle pacing; HF: heart failure; LBBAP: left bundle branch area pacing; RV: right ventricular; SD: standard deviation.

## Data Availability

The data presented in this study are available on request from the corresponding author.
